# Mapping malaria seasonality in Madagascar using health facility data

**DOI:** 10.1186/s12916-019-1486-3

**Published:** 2020-02-10

**Authors:** Michele Nguyen, Rosalind E. Howes, Tim C.D. Lucas, Katherine E. Battle, Ewan Cameron, Harry S. Gibson, Jennifer Rozier, Suzanne Keddie, Emma Collins, Rohan Arambepola, Su Yun Kang, Chantal Hendriks, Anita Nandi, Susan F. Rumisha, Samir Bhatt, Sedera A. Mioramalala, Mauricette Andriamananjara Nambinisoa, Fanjasoa Rakotomanana, Peter W. Gething, Daniel J. Weiss

**Affiliations:** 1 Malaria Atlas Project, Oxford Big Data Institute, Nuffield Department of Medicine, University of Oxford, Oxford, UK; 2 Department of Infectious Disease Epidemiology, Imperial College London, London, UK; 3 National Malaria Control Programme, Antananarivo, Madagascar; 4 Unité d’Epidémiologie, Institut Pasteur de Madagascar, Antananarivo, Madagascar

**Keywords:** Seasonality, Malaria, Geostatistical model, Health facility data, Madagascar

## Abstract

**Background:**

Many malaria-endemic areas experience seasonal fluctuations in case incidence as *Anopheles* mosquito and *Plasmodium* parasite life cycles respond to changing environmental conditions. Identifying location-specific seasonality characteristics is useful for planning interventions. While most existing maps of malaria seasonality use fixed thresholds of rainfall, temperature, and/or vegetation indices to identify suitable transmission months, we construct a statistical modelling framework for characterising the seasonal patterns derived directly from monthly health facility data.

**Methods:**

With data from 2669 of the 3247 health facilities in Madagascar, a spatiotemporal regression model was used to estimate seasonal patterns across the island. In the absence of catchment population estimates or the ability to aggregate to the district level, this focused on the monthly proportions of total annual cases by health facility level. The model was informed by dynamic environmental covariates known to directly influence seasonal malaria trends. To identify operationally relevant characteristics such as the transmission start months and associated uncertainty measures, an algorithm was developed and applied to model realisations. A seasonality index was used to incorporate burden information from household prevalence surveys and summarise ‘how seasonal’ locations are relative to their surroundings.

**Results:**

Positive associations were detected between monthly case proportions and temporally lagged covariates of rainfall and temperature suitability. Consistent with the existing literature, model estimates indicate that while most parts of Madagascar experience peaks in malaria transmission near March–April, the eastern coast experiences an earlier peak around February. Transmission was estimated to start in southeast districts before southwest districts, suggesting that indoor residual spraying should be completed in the same order. In regions where the data suggested conflicting seasonal signals or two transmission seasons, estimates of seasonal features had larger deviations and therefore less certainty.

**Conclusions:**

Monthly health facility data can be used to establish seasonal patterns in malaria burden and augment the information provided by household prevalence surveys. The proposed modelling framework allows for evidence-based and cohesive inferences on location-specific seasonal characteristics. As health surveillance systems continue to improve, it is hoped that more of such data will be available to improve our understanding and planning of intervention strategies.

## Background

Malaria is a disease caused by the *Plasmodium* parasite and remains a major cause of child mortality in sub-Saharan Africa [1]. As with many vector-borne diseases, malaria transmission exhibits seasonality across endemic areas. That is, malaria burden, which can be measured by metrics including parasite prevalence or the number of clinical cases, follows an annually recurring seasonal pattern that is typically attributed to the relationship of the mosquito vector and parasite life cycles with the environment. The rationale for developing methods capable of enumerating location-specific seasonal characteristics is to assist planning for intervention distributions to improve their efficacy, develop early warning systems, and improve the temporal resolution and overall accuracy of malaria burden estimation models [2].

Past studies on the seasonality of malaria vary in their degree of sophistication and scope. For example, some give empirical descriptions of the cyclic patterns at specific locations, while others model the time series by relating them to underlying seasonal conditions or mathematical structures such as in seasonal autoregressive integrated moving average models and trigonometric models [3–8].

To guide intervention policies, there have also been attempts to derive maps related to seasonality. By using thresholds of, for example, rainfall, temperature, and vegetation cover, it is possible to estimate the start, the end, and the length of the period suitable for transmission [9–11]. Maps of malaria seasonality patterns are relevant to the planning of intervention campaigns to maximise their impact. For example, seasonal malaria chemoprevention (SMC) has been shown to be most effective when delivered in areas with highly seasonal transmission. As such, the World Health Organization guidelines recommend targeted SMC in malaria endemic areas where more than 60% of clinical cases occur during a short period of about 4 months [12].

Despite their potential utility, the threshold-based malaria seasonality maps have several limitations. For example, metrics such as total rainfall can be linked to either the creation or the washing away of mosquito breeding sites depending on the local topology and rainfall intensity [13, 14]. Using fixed environmental thresholds does not account for the variation of responses to environmental forcing or allow for other potential drivers such as seasonal migration of human populations [15, 16]. Likewise, because the distribution of vector species is spatially heterogeneous and their preferences for breeding sites vary, a one-size-fits-all approach for characterising malaria seasonality may miss important nuances [17].

Another class of seasonality maps consists of concentration indices that aim to quantify the proportion of an annual amount (of any variable of interest) which falls within a subannual window of fixed size. In order to quantify the distribution of malaria cases in each district over a year, Mabaso et al. used Markham’s concentration index, a measure previously used to determine rainfall concentrations [18]. In their analysis, the concentration maps from the case numbers that were estimated using a Bayesian spatiotemporal regression model displayed clearer spatial patterns compared to those derived from raw case numbers. Spatiotemporal models smooth out idiosyncratic deviations, thus enabling the main seasonal trend to be discerned more easily. They are also useful for explicitly relating the seasonality to input covariates as well as accounting for unknown spatiotemporal effects.

In this paper, we build on previous attempts and map malaria seasonality in Madagascar, a country of marked ecological and epidemiological diversity that is struggling to meet targets for malaria burden reductions and hence where further information for optimising interventions would be valuable [1, 19]. We demonstrate how common descriptors of seasonality can be made comparable across locations to facilitiate modelling and support policy decisions, a topic of interest for not just modellers but also the wider public health community. The modelling framework, which aims to provide a cohesive and evidence-based analysis, is applied to 2013–2016 health facility case data from Madagascar. Despite the lack of catchment population estimates or data from all health facilities, we show that the monthly case data can be used to model spatially heterogenous seasonal patterns of malaria. We identify seasonality features such as the peak and length of transmission by estimating the proportions of cases in each month using a log-linear spatiotemporal regression model and fits to rescaled von Mises densities. By applying the algorithm to posterior samples of the monthly proportion curves, we also obtain uncertainty measures associated with each derived seasonal characteristic. To reflect both the timing and the amplitude aspects of seasonality, a seasonality index is used to synthesise the monthly case proportion estimates with existing annual case or parasite incidence (API) estimates.

## Methods

### Study data

Malaria seasonality in Madagascar was modelled using monthly case reports submitted by health facilities to the centralised Ministry of Health between 2013 and 2016. This dataset was provided by the National Malaria Control Programme (NMCP) in Madagascar and gave the number of patients who tested positive for malaria by rapid diagnostic tests (RDTs), irrespective of species. After cleaning the data to ensure consistent nomenclature, 2801 health facilities were successfully geo-located and verified using a database of 3274 geo-located health facilities from the Institut Pasteur de Madagascar (IPM). The IPM database itself was further validated against a database of 120 global positioning system (GPS) geo-located health facilities from the President’s Malaria Initiative (PMI).

**Fig. 1:**
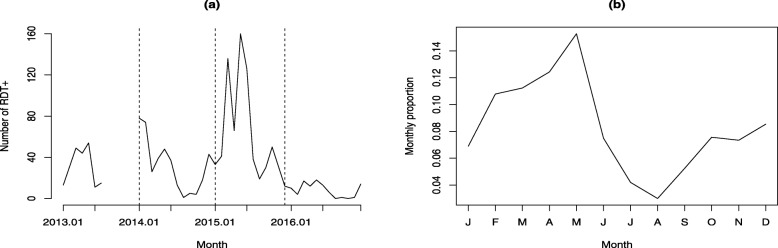
**a** Number of positive rapid diagnostic tests (RDTs) reported from an example Malagasy health centre recorded monthly between 2013 and 2016. **b** The corresponding monthly proportions computed by dividing the monthly medians by the annual total

To account for year-on-year trends and help avoid unwanted bias in the monthly health facility data from, for example, stock-outs of RDTs, median monthly case counts were derived for each site. With a focus on location-specific seasonal trends, monthly proportions were obtained by dividing the monthly case medians by their annual sum. This is illustrated for an example Malagasy health centre in Fig. 1. After excluding sites with incomplete intra-annual patterns, data from 2669 health facilities were used in the later analysis.

By focusing on proportions instead of the case counts, we bypass the need to estimate catchment populations for the health facilities. Deriving incidence measures using catchment population data is commonly used to adjust for magnitude differences in count data, but estimating these catchment population sizes could have introduced bias to the analysis as only an incomplete set of health facility geolocation data was available to inform this analysis. Additional features of this modelling approach include the following: (a) utilising a standardised definition of a transmission period as the months with the proportion of cases exceeding 1/12 of the annual total, and (b) restricting the analysis to dynamic environmental covariates like rainfall and temperature that are likely to impact seasonal malaria transmission patterns.

A suite of environmental variables were assembled as 5 km spatial grids. These were temporally aligned with the health facility data and included the Climate Hazards Group Infrared Precipitation and Station data (CHIRPS), enhanced vegetation index (EVI), daytime land surface temperature (LST_day), diurnal difference in land surface temperature (LST_delta), night-time land surface temperature (LST_night), tasselled cap brightness (TCB), tasselled cap wetness (TCW), and the temperature suitability indices for *Plasmodium falciparum* and *Plasmodium vivax* (TSI_Pf and TSI_Pv). Details on the sources of these data are available elsewhere [19, 20]. To relate the observed seasonal patterns to the potential driving factors, monthly medians of these environmental data were derived and standardised. One to 3 month lags were included for each covariate to allow for delayed and accumulated responses to these environmental variables [19].

### Spatiotemporal monthly proportion model

Proportions or probabilities are often modelled using multinomial or compositional regressions. However, in this scenario, this would mean computing ratios with respect to a fixed reference month. By using monthly case proportions, it is easier to relate each month’s proportion to the values of its covariates explicitly. To estimate monthly case proportions over the study region, we use the following spatiotemporal model which can be viewed as the linear predictor of a multinomial regression written in a log-linear form:

1 $$ \log(p_{i,j}) = \boldsymbol{X}_{ij}\boldsymbol{\beta} + \phi_{ij} + \epsilon_{ij}.   $$

Here, *p*_*i*,*j*_ is the average proportion of cases at location *j* in month *i*. To avoid applying logarithms on zeros, we added an offset of 0.00001 to *p*_*i*,*j*_ and rescaled the raw monthly proportions at each location to sum to one before modelling. Similarly, rescaling was conducted after modelling with a location-specific normalising constant. This allows locations to be more or less sensitive to the variation in the underlying covariates.

In Eq. (1), $\boldsymbol {X}_{ij}\in \mathbb {R}^{n\times m}$ is a covariate design matrix including an intercept, $\boldsymbol {\beta }\in \mathbb {R}^{m}$ is the corresponding parameter vector, and $\epsilon \sim N(0, \sigma _{e}^{2})$ denotes the independent, identically distributed noise. The spatiotemporal Gaussian field *ϕ* is constructed such that:

2 $$ \phi_{i,j} = \left\{\begin{array}{l} \xi_{1j} \text{ for}\, i = 1, \\ a \phi_{i-1,j} + \xi_{i, j} \text{ for} \, i=2, \dots, 12, \end{array}\right.   $$

|*a*|<1 and *ξ*_*i*,*j*_ correspond to zero-mean Gaussian innovations which are temporally independent but spatially coloured with a Matérn covariance:

3 $$ \text{Cov}(h) = \frac{\sigma^{2}_{f}}{\Gamma(\nu)2^{\nu-1}}(\kappa h)^{\nu} K_{\nu}(\kappa h),   $$

where *h* is the distance between two locations and *κ*>0 is a scaling parameter. In practice, it is difficult to identify the order of the modified Bessel function of second kind, denoted by *K*_*ν*_ in Eq. (3) [21]. Thus, this was set to 1 as per the convention in the R-INLA package, which was used for model fitting and selection [22–24].

Before fitting the model, the log proportions were examined and outliers were excluded to model prototypical seasonal behaviour. Based on the histogram of log(*p*_*i*,*j*_) values in Additional file 1: Figure S1, log(*p*_*i*,*j*_)≤− 11 were deemed as outliers. Since the proportions were previously rescaled to sum up to one at each location, this does not mean that all data zeros were excluded from the analysis. Instead, zeros were only removed if much higher case proportions were observed in other months.

A randomly selected 30% of sites were excluded from the model fitting to validate our results. Working with data from the remaining 70% of the locations, the set of covariates was reduced to facilitate model selection and account for multicollinearity by iteratively computing the variance inflation factors (VIFs) and removing the covariates with the highest VIF value until all the remaining covariates have VIF values less than 10. Since these covariates have low correlations with each other, their estimated regression coefficients are more robust.

To speed up the covariate selection via backwards regression, the 70% training set was randomly split into two smaller sets of equal size and spatial coverage to search for the best model in terms of deviance information criterion (DIC). A map of the test and training locations is shown in Additional file 1: Figure S2. The two resulting candidates from the separate backwards regressions on the two smaller training sets were then refitted to the whole training set to select the final model with the lower DIC. After checking for reasonable results on the test data, the model was refitted to the entire dataset before predictions were made over the gridded surface. An analysis of the robustness of the methodology to the spatial and temporal extents as well as the quality of the data is provided in the Additional file 1.

### Seasonality index and monthly case incidence

Seasonality statistics were derived from each posterior sample of the location-specific monthly proportions. To quantify ‘how seasonal’ a location is, a seasonality index was defined [25]. For location *j*, this index is given by the product of an entropy measure and the normalised amplitude:

4 $$\begin{array}{*{20}l} S_{j} &= D_{j} \times \frac{R_{j}}{R_{\text{max}}},  \end{array} $$

5 $$\begin{array}{*{20}l} \text{where}\, D_{j} &= \sum_{i = 1}^{12} p_{i,j} \log_{2}\left(\frac{p_{i,j}}{q}\right). \end{array} $$

Since *p*_*i*,*j*_ is the case proportion for month *i* and *q*=1/12, *D*_*j*_ corresponds to the Kullback–Leibler divergence between the estimated intra-annual distribution and a uniform distribution. Thus, it quantifies how different the monthly proportions are from a uniform distribution over the year. In the context of malaria, *R*_*j*_ can be represented by the API at location *j* and *R*_max_ is the maximum API over the region.

One benefit of using *S*_*j*_ is that it separates the timing and amplitude aspects of seasonality. Since high-resolution malaria burden estimates already exist [26–28], the model did not need to estimate the number of cases and could focus exclusively on estimating the monthly proportions at each location. Estimates of the monthly parasite incidence (MPI) for each location could also be obtained by multiplying the estimated monthly proportions with the mean *Pf* API estimates for 2016. This synthesises the estimated seasonal pattern obtained from the health facility data with the magnitude-level information provided by household prevalence surveys since these were used in the API model.

### Deriving seasonality features

Locations were considered as potentially seasonal if their entropy *D*_*j*_>0. When this criterion was satisfied, a rescaled von Mises (RvM) density was fitted via least squares to the estimated monthly proportions. This is illustrated for an example Malagasy health facility in Additional file 1: Figure S3.

By treating the month in a year as a random variable on a circle, i.e. defining $\theta = \frac {2\pi i}{12}$ where *i*=1,…,12, the two-component RvM density function can be written as follows:

6 $$ \begin{aligned} f(\theta; s, \omega, \mu_{1}, \kappa_{1}, \mu_{2}, \kappa_{2}) &= s\left[\omega f_{1}(\theta; \mu_{1}, \kappa_{1})\right. \\&\quad\left.+ (1-\omega) f_{2}(\theta; \mu_{2}, \kappa_{2})\right] \end{aligned}  $$

7 $$ \begin{aligned} \text{where}\, f_{k}(\theta; \mu_{k}, \kappa_{k}) &= \frac{1}{2\pi I_{0}(\kappa_{k})} \exp\{\kappa_{k}\cos(\theta - \mu_{k})\}  \end{aligned}  $$

is a one-component vM density for *k*=1,2 with mean and concentration parameters *μ*_*k*_ and *κ*_*k*_. Here, *I*_0_ is the modified Bessel function and *ω* is a probability weight. The scale parameter *s*>0 modulates between the continuous density function and monthly proportions over discrete months.

Instead of identifying characteristics based on the monthly proportion estimates directly, seasonal features were based on fitted vM curves, which further smoothed out the estimates. The benefit of using a circular distribution was the continuity of the curve between the months of December and January. Using vM densities, in particular, is convenient for identifying the peaks of the distribution since these correspond to the mean parameters [29]. By comparing the values of the fitted curve, the major and the minor peaks of a bimodal distribution can be identified. Although an arbitrary number of von Mises components can be used, one or two were used because areas with seasonal malaria transmission typically have one or two main seasons [2].

To reduce computational burden, a bimodal distribution was only considered if the error from the fit of a unimodal distribution exceeded a set threshold $\tilde {\epsilon }>0$. For Madagascar, $\tilde {\epsilon }$ was empirically chosen to be 0.0015. Based on the fitted vM curve, the transmission periods were identified by marking the months where the curve was at or above $\frac {1}{12}$. In this way, the start, end, and length of each season could also be estimated. Algorithm 1 summarises the procedure used to obtain the seasonality statistics from the monthly proportion realisation curves.

To quantify the uncertainty associated with the derived statistics, the results from 100 posterior samples of the monthly proportions were summarised. A location was deemed as unimodal or bimodal if more than half of the samples supported that interpretation and the degree of certainty was the proportion of such samples. Based on this majority decision and the corresponding samples, the uncertainty was also assessed in the estimated seasonal characteristics. Circular medians and deviations were used for the start, end, and peak of each transmission season [30]. To interpret the deviations in terms of months, the circular deviations was multiplied by $\frac {12}{2\pi }$.


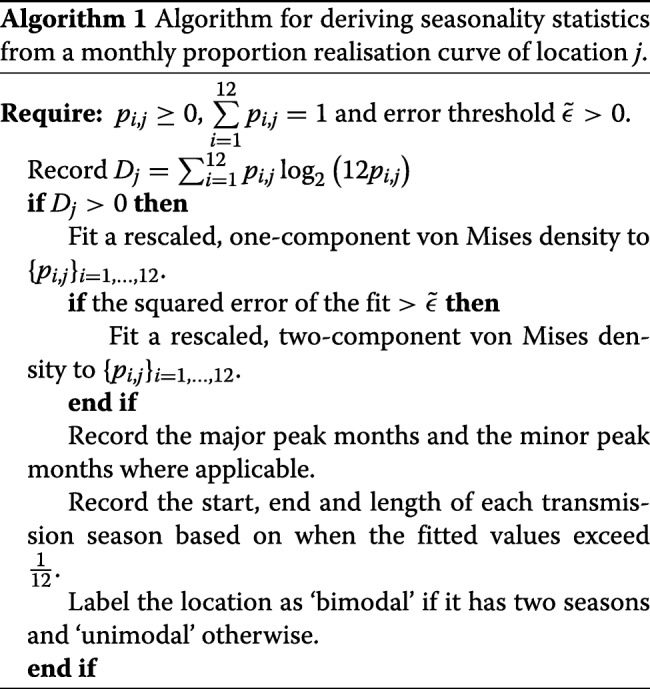


## Results

### Dominant environmental relationship

The regression component of the log-linear spatiotemporal model allows us to infer the dominant relationship between the monthly case proportions and the environmental covariates while the spatiotemporal random field accounts for deviations from this. As expected, the selected model (Table 1) suggests positive relations between the monthly case proportions and rainfall for the concurrent month as well as at 2- and 3-month lags [20, 31, 32]. There is also a positive relation with the *Plasmodium vivax* temperature suitability index at a 2-month lag. The latter is a modelled parameter that estimates the combined effect of temperature on *Anopheles* survival as well as the development of sporozoites within mosquitoes. A high temperature suitability value indicates that many mosquitoes will survive long enough to become infectious. Since this index was derived from a biological model, it accounts for a non-linear relationship with the monthly proportions.

**Table 1:** Parameter posterior summaries of the refitted model for Madagascar

Description	Term	Posterior median	95% credible interval
Intercept	Intercept	*−* *2*.*655*	(− 2.740,− 2.570)
Precipitation	CHIRPS_r	*0*.*060*	(0.029,0.090)
	CHIRPS_r_lag1	0.019	(− 0.012,0.050)
	CHIRPS_r_lag2	*0*.*051*	(0.021,0.082)
	CHIRPS_r_lag3	*0*.*053*	(0.022,0.084)
Temperature suitability	TSI_Pv_r	0.013	(− 0.022,0.047)
	TSI_Pv_r_lag2	*0*.*074*	(0.039,0.109)
Vegetation cover	EVI_r_lag3	0.006	(− 0.021,0.032)
Tasselled cap brightness	TCB_r	− 0.019	(− 0.050,0.012)
	TCB_r_lag3	0.011	(− 0.020,0.043)
Observation variance	$\sigma _{e}^{2}$	0.326	(0.318,0.333)
Field variance	$\sigma _{f}^{2}$	0.245	(0.221,0.268)
Matérn scaling parameter	*κ*	3.163	(2.834,3.522)
Autoregressive parameter	*a*	*0*.*756*	(0.718,0.777)

The posterior medians of the statistically significant parameters under a 5% significance level are italicized. The Matérn smoothness parameter *ν* was fixed to 1

### Seasonality categories and monthly parasite incidence estimates

**Fig. 2:**
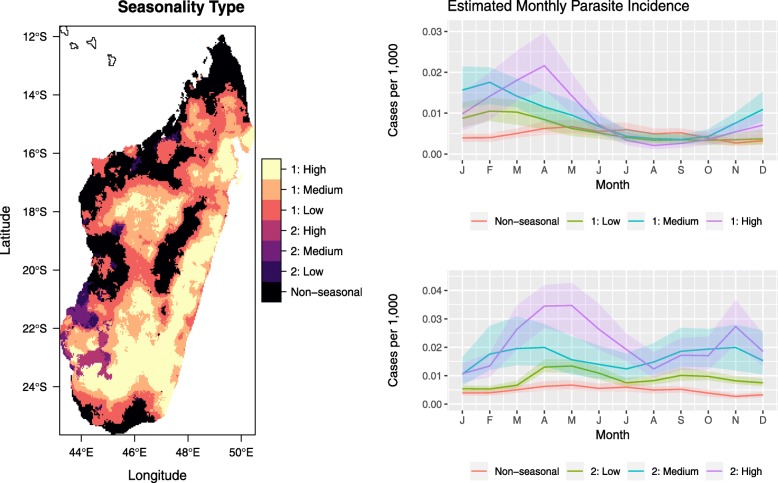
Map of seasonality types based on quartiles of the estimated seasonality index as well as representative examples of the estimated monthly parasite incidence for the categories. Here, ‘1’ and ‘2’ refer to the unimodal and bimodal intra-annual distributions, respectively, while ‘Low’, ‘Medium’, and ‘High’ refer to the different degrees of seasonality

‘How seasonal’ malaria is in a location is related to both the magnitude and the intra-annual distribution of cases. This is quantified using the seasonality index. Figure 2 shows the map of seasonal types derived from the median seasonality index, as computed using 100 realisations from the fitted model for Madagascar. The different degrees of seasonality (‘Non-seasonal’, ‘Low’, ‘Medium’, and ‘High’) were defined for the unimodal and bimodal locations separately using the quartiles of their seasonality indices.

Examples of the estimated MPI curves for each seasonal category are shown alongside the map. In general, we observe that higher seasonality indices are associated with higher average levels of MPI as well as greater amplitudes of the fluctuations. As shown in Additional file 1: Figure S4, the bimodal locations (i.e. those with two seasonal peaks in MPI) tend to have lower seasonal index values than the unimodal locations because their distributions are more spread out over the year.

### Seasonal features and associated uncertainties

Next, we focus on the timing aspect of seasonality and derive seasonality characteristics such as the start and peak months of transmission. Since we work with the estimated monthly case proportions which rely on the environmental covariates and spatiotemporal correlation, we also obtain results for areas deemed ‘non-seasonal’ via the seasonal index in Fig. 2. Following the definition of the seasonality index in Eq. (4), such non-seasonality could arise due to a relatively uniform intra-annual distribution of cases or extremely low malaria burden. In the latter scenario, the derived seasonality features describe a theoretical transmission season which could materialise if transmission re-establishes itself.

**Fig. 3:**
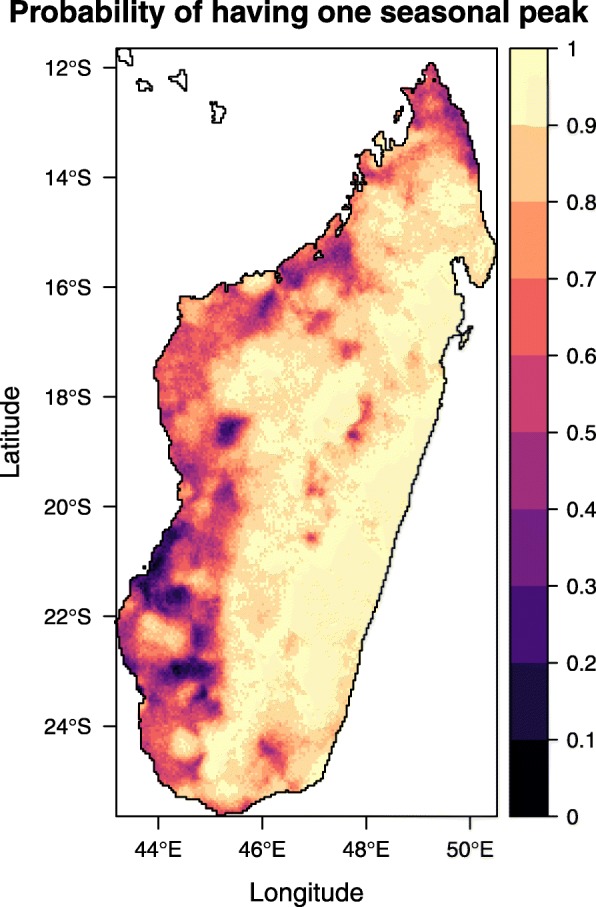
Probability of locations having one seasonal peak in malaria cases. This is calculated by the proportion of posterior samples which indicate that the locations have unimodal intra-annual case distributions rather than bimodal distributions

From Fig. 2, we see that most of the island was deemed to have unimodal seasonality. However, as shown in Fig. 3, there is generally less certainty along the western and northern coasts. This is consistent with the analysis of Liebmann et al. which described increased likelihood of bimodal rainfall patterns on the west coast and unimodal trends on the east coast [33]. The lower data availability (see Additional file 1: Figure S2) as well as the remoteness of western and northern coasts could also contribute to the uncertainty of the estimates in these regions [34]: lower reporting rates and differing care-seeking behaviour, which could arise due to the lower accessibility of the health facilities, can cause conflicting seasonal signals in the data.

**Fig. 4:**
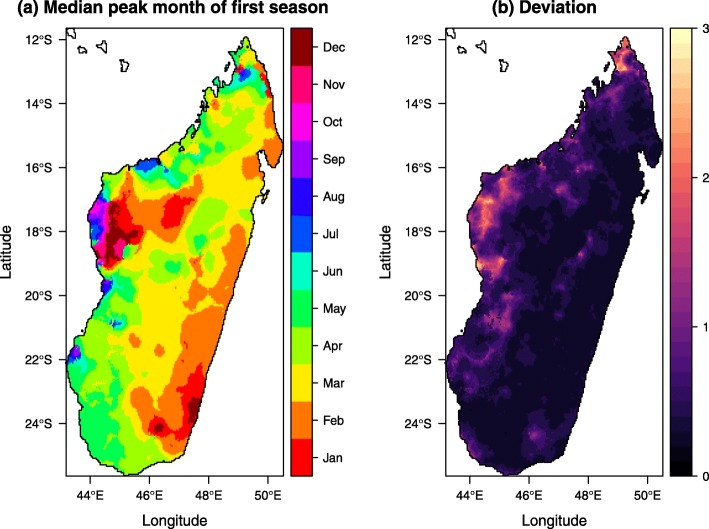
**a** Median peak months of the first transmission season in Madagascar. **b** The associated deviations

The median peak months and associated deviations of the first transmission season are shown in Fig. 4. The results are consistent with the existing literature [34]. Large parts of the island experience peaks in March–April while the east coast sees an earlier peak around February. The heterogeneity in Fig. 4a in the western region of Melaky (near 45^∘^ E, 17^∘^ S) is associated with high deviations. This may be due to its remoteness and low population density [34]. In Additional file 1: Figure S5, we show the time series of the number of people tested positive for malaria via RDTs between 2013 and 2016 at three example health facilities in Melaky. The relatively low and highly variable case numbers lead to higher stochasticity in the observed and estimated seasonality patterns. Reporting difficulties, as illustrated by the multiple gaps in the time series, add further uncertainty to the derived monthly proportion curves. Additional seasonality plots including the maps of transmission end months and transmission season duration can be found in the Additional file 1.

## Discussion

This paper introduces a statistical modelling framework for mapping malaria seasonality in Madagascar using health facility data. The approach relies on a log-linear spatiotemporal regression model to smooth and estimate location-specific monthly proportions of cases. As countries increasingly adopt digital surveillance platforms such as the District Health Information Software 2 (DHIS2) for the digital recording of cases at the health facility level, it is hoped that more of such data will be available to inform seasonal and localised intervention strategies.

Due to the nature of the health facility data, only relative levels of burden can be derived to inform location-specific seasonal trends. The modelling framework leverages existing API maps to bring together the amplitude as well as timing aspects of seasonality. For a cohesive analysis, characteristics such as the start, peak, and length of each transmission season as well as MPI estimates are obtained via the same estimated curve of monthly proportions. The latter is also used to compute a seasonality index and categorise seasonality types. For each seasonal feature, measures of uncertainty are also presented to facilitate statistically sound decision-making.

**Fig. 5:**
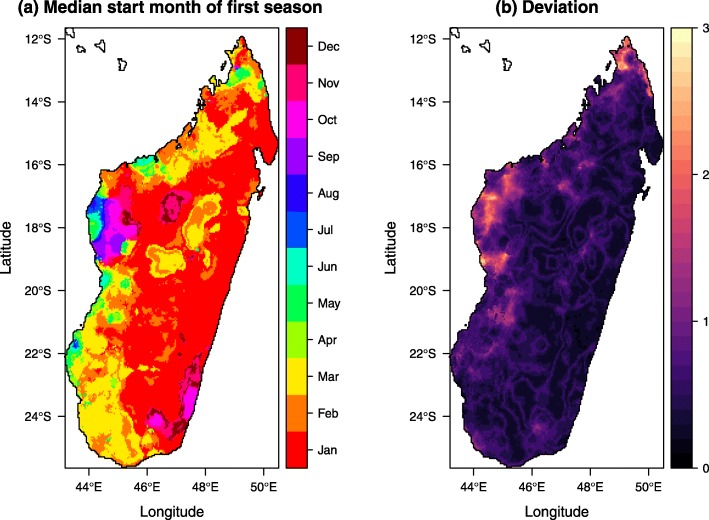
**a** Median start months of the first transmission season in Madagascar. **b** The associated deviations

In Madagascar, the 2018–2022 National Strategic Plan includes indoor residual spraying (IRS) as a tool to help reduce transmission in the highest disease burden districts and as an emergency response tool to epidemic outbreaks [35, 36]. Given that sprayed insecticide generally remains efficacious for less than 6 months (depending on the insecticide used and types of surfaces sprayed)[37], local seasonality patterns are important to guide optimal timing of IRS campaigns. Spraying must be timed for completion ahead of the start of transmission, but not so early that the insecticide bio-efficacy will wear off before the end of the season. Through this modelling framework, we can estimate the median start months and associated deviations of the first transmission season. The results, as illustrated in Fig. 5, suggest that IRS should be completed in southeast districts ahead of the southwest. This is in line with PMI’s 2017–2018 strategy.

Despite the many advantages of this approach, there are some limitations. An important assumption was made when the empirical monthly proportions were computed by averaging case counts over a number of years. The notion that there should be a static seasonal trend over multiple years is a common and practical one; however, while climatic patterns are broadly predictable, there is significant inter-annual variability in factors such as the beginning of the rainy season. The impact of the annual cyclone season in Madagascar is particularly significant in this respect, triggering both unusually high rainfall (and subsequently increased mosquito vector abundance) and infrastructure damage which can severely disrupt malaria control intervention efforts, resulting in unusual patterns of malaria outbreaks [34, 38]. Likewise, periodic events related to El Niño-Southern Oscillation and/or global climate change also impact malaria seasonal patterns [31]. Changes in vector species composition and behaviour driven by large-scale coverage of vector control interventions (such as the 2013 and 2015 national campaigns to distribute insecticide-treated nets) could potentially also result in shifts to the timings of the transmission season [39]. For example, a novel vector, *Anopheles coustani*, was recently described in the Malagasy highlands [40], and evidence of strong, fine-spatial scale differences in vector behaviour also allows for adaptive plasticity in response to external pressures which could translate to changes in parasite transmission over time [41]. Given this reality, future iterations of this work could use a moving window approach to continually update the model with new data.

**Fig. 6:**
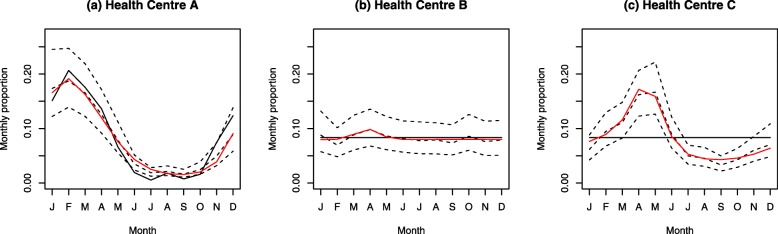
Examples of the model fit and rescaled von Mises density fit for three health facilities. The black line denotes the empirical monthly proportions of cases, the black dotted lines represent the median proportions and 95% credible intervals, and the red line the fitted rescaled von Mises density. Note that no cases were reported for Health Centres B and C, leading to a uniform empirical intra-annual distribution. **a** Health Centre A. **b** Health Centre B. **c** Health Centre C

Another noteworthy limitation of the proposed methodology relates to relying on sparse, spatially discrete health facility reports to establish seasonal patterns across space. Although the issue of under-reporting due to RDT stock-outs was mitigated somewhat by averaging case counts over several years, the issue of zero recorded cases in a facility still poses a potential problem as these could be due to the low parasite prevalence (whether historical or only recent) or the low popularity of the facility itself. Another consideration is whether a zero is a true representation of cases in a facility rather than a placeholder for unreported data. While we have tried to ensure the accuracy of the response data by, for example, omitting health facilities with insufficient data to establish a full year-long seasonal trend, it is possible that flawed points were included in the model. This possibility is illustrated in Fig. 6, which shows the empirical and fitted monthly proportions for three health facilities. Health Centre B (Fig. 6b) illustrates a scenario where there were no cases and the model estimated a non-seasonal trend. In contrast, Health Centre C (Fig. 6c) had no cases but an estimated seasonal trend. The clearest interpretation of a pattern in the absence of data is that while there were no cases reported, the environmental conditions in that area suggest that if there were to be any cases, they would tend to peak in April. Such an interpretation is analogous to the theoretical peak transmission season in a country that has eliminated malaria, yet continues to experience seasonally high vector densities.

The presented model establishes the dominant relationship between environmental covariates and monthly proportions in the study area. This is driven by data in obviously seasonal locations such as Health Centre A in Fig. 6a. The spatiotemporal field accounts for other unknown factors and smooths out the estimated monthly proportions between locations. In this way, information is borrowed from the seasonal locations identified within the data and applied to areas with similar environmental profiles.

In addition to the level of malaria burden, spatial scale affects the amount of stochasticity in seasonality analyses. Although we bypassed the issue of catchment populations by modelling monthly proportions instead of case numbers, the number of cases seen at a health facility will be more variable if it serves less people. This was seen for the Melaky region in the Madagascar case study. If we had data for all health facilities and aggregated the cases to the administrative (district) level, it is likely that a stronger seasonal signal would be observed. The trade-off is that the relation between administrative-level seasonality and area-representative environmental covariates (e.g. average rainfall) may be less strong.

The seasonality we model is limited by the nature of our data and the available seasonal signal as well as the relations we can establish. Since we work with case data, if treatment-seeking behaviour itself is seasonal and related to environmental factors such as rainfall, the seasonality we observe and hence model is merely the seasonality of cases at health facilities which may not be reflective of the seasonal trends for cases at the population level.

As previously mentioned, different settings can give rise to different responses to environmental forcing. For example, while one frequently links increased mosquito breeding habitats to the period after the rainy season, in the Brazilian Amazon, this is instead linked to the dry season when small, isolated water bodies are created with the receding of rivers [13, 14]. While the spatiotemporal field in our model helps adjust for differences from the dominant environmental relation, more research is required on these different settings and how to integrate them into our model structure. Local knowledge may also be useful for adjusting the models to, for example, subset the study regions based on differing responses.

## Conclusion

Malaria seasonality maps are useful for targeting interventions such as seasonal malaria chemoprevention and indoor residual spraying. As illustrated for Madagascar, subannual health facility data can be used to establish seasonal patterns in malaria burden and augment the information provided by household prevalence surveys. The proposed modelling framework represents a robust approach towards obtaining evidence-based seasonality maps and estimates. With the ability to infer the dominant environmental relation in the study region as well as to provide cohesive results and uncertainty measures for the estimated seasonal features, this research presents an advancement from the existing threshold and concentration-based mapping procedures. As more health facility case data becomes available, it is hoped that more of such data will be available to improve our understanding and planning of intervention strategies.

## Supplementary information

**Additional file 1** Supplementary information including additional illustrations of the methodology, time series and empirical monthly proportion curves for three example health centres in Melaky as well as maps of other estimated seasonal characteristics such as the lengths of the transmission seasons. This document also contains an additional analysis of the methodology’s robustness towards changes in data quality as well as temporal and spatial extents.

## Data Availability

Sample, anonymised data, and sample code are available at http://bitbucket.org/mntd/malaria-seasonality. The raw data that support the findings of this study are available from the Programme National de Lutte Contre le Paludisme de Madagascar and the Institut Pasteur de Madagascar (IPM). Health facility locations were updated and prepared by the SaGEO (Santé et GEOmatique) group at IPM. The source was made available to IPM by the Service d’Information Sanitaire(SIS)/Ministry of Health.

## Abbreviations

API: Annual parasite incidence
CHIRPS: Climate Hazards Group Infrared Precipitation and Station
DHIS2: District Health Information Software 2
DIC: Deviance information criterion
EVI: Enhanced vegetation index
GPS: Global positioning system
IPM: Institut Pasteur de Madagascar
IRS: Indoor residual spraying
LST: Land surface temperature
MPI: Monthly parasite incidence
NMCP: National Malaria Control Programme
Pf: *Plasmodium falciparum*
PMI: President’s Malaria Initiative
Pv: *Plasmodium vivax*
RDT: Rapid diagnostic test
RvM: Rescaled von Mises
SMC: Seasonal malaria chemoprevention
TCB: Tasselled cap brightness
TCW: Tasselled cap wetness
TSI: Temperature suitability index
VIF: Variance inflation factor
