# Supplementary material for: Mapping malaria seasonality in Madagascar using health facility data
Source: BMC Med. 2020 Feb 10;18:26. doi: 10.1186/s12916-019-1486-3 (PMC7008536; doi:10.1186/s12916-019-1486-3)
Supplement: Supplementary file 1 — Additional file 1 Supplementary information including additional illustrations of the methodology, time series and empirical monthly proportion curves for three example health centres in Melaky as well as maps of other estimated seasonal characteristics such as the lengths of the transmission seasons. This document also contains an additional analysis of the methodology’s robustness towards changes in data quality as well as temporal and spatial extents. [file 12916_2019_1486_MOESM1_ESM.pdf]

Supplementary information for "Mapping malaria seasonality in Madagascar using health facility data"

Michele Nguyen[1*], Rosalind E. Howes[1], Tim C.D. Lucas[1], Katherine E. Battle[1], Ewan Cameron[1], Harry S. Gibson[1], Jennifer Rozier[1], Suzanne Keddie[1], Emma Collins[1], Rohan Arambepola[1], Su Yun Kang[1], Chantal Hendriks[1], Anita Nandi[1], Susan F. Rumisha[1], Samir Bhatt[2], Sedera A. Mioramalala[3], Mauricette Andriamananjara Nambinisoa[3], Fanjasoa Rakotomanana[4], Peter W. Gething[1†], Daniel J. Weiss[1†]

[1]Malaria Atlas Project, Oxford Big Data Institute, Nuffield Department of Medicine, University of Oxford, Oxford, UK. *Corresponding author: M. Nguyen (michele.nguyen@bdi.ox.ac.uk). † Joint senior authors.

[2]Department of Infectious Disease Epidemiology, Imperial College London, London, UK.

[3]National Malaria Control Programme, Antananarivo, Madagascar.

[4]Unité d'Epidémiologie, Institut Pasteur de Madagascar, Antananarivo, Madagascar.

S1 Additional figures

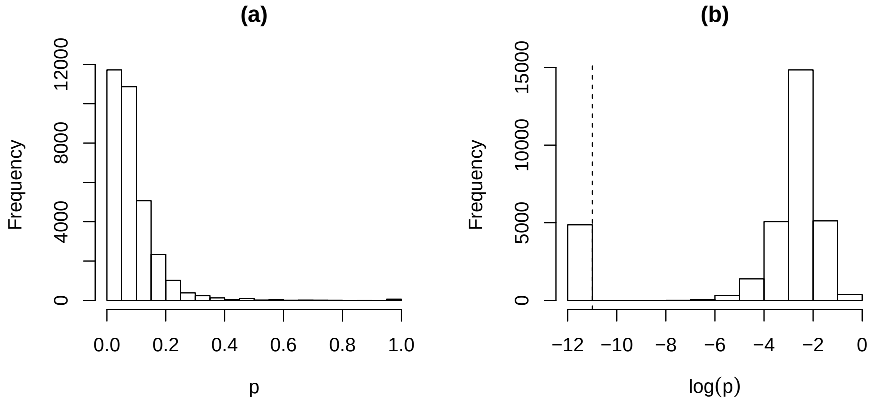

Figure S1: (a) Histogram of the empirical monthly proportions ($p_{i,j}$) from the Madagascar health facility data and (b) histogram of the log-transformed monthly proportions ($\log(p_{i,j})$). Since points with $\log(p_{i,j}) \leq -11$ are treated as outliers, the dotted vertical line in (b) denotes cut-off.

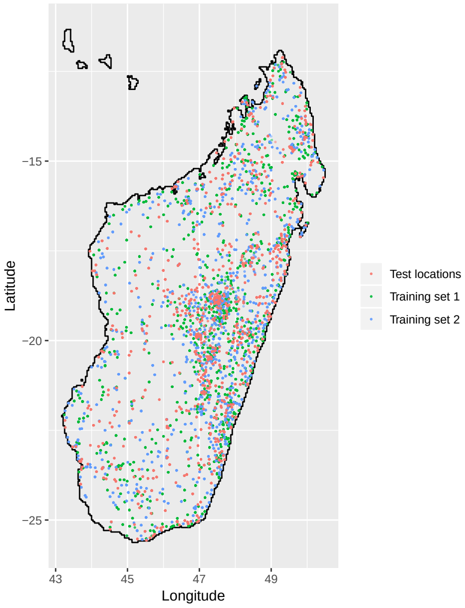

Figure S2: Locations of the test and training health facilities for Madagascar.

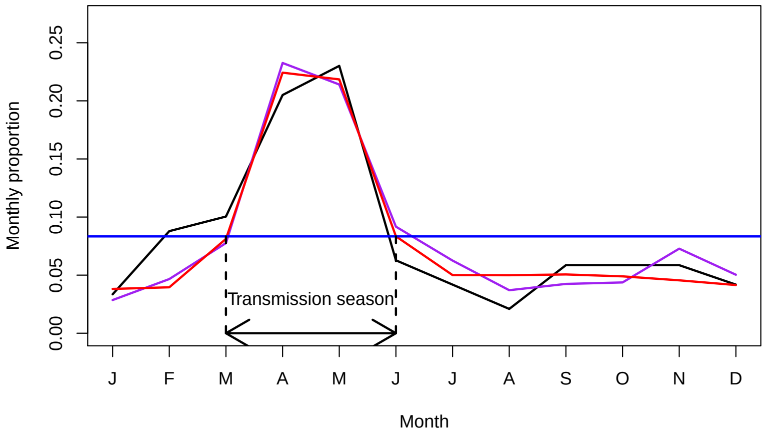

Figure S3: An illustration of how the seasonal characteristics can be identified from monthly proportion realisations for an example Malagasy health centre. The empirical proportions are denoted by the black line while one posterior sample is given in purple and its fitted rescaled von Mises density is given in red. The horizontal blue line denotes the $1/12$ threshold and the dotted lines mark out the derived transmission season.

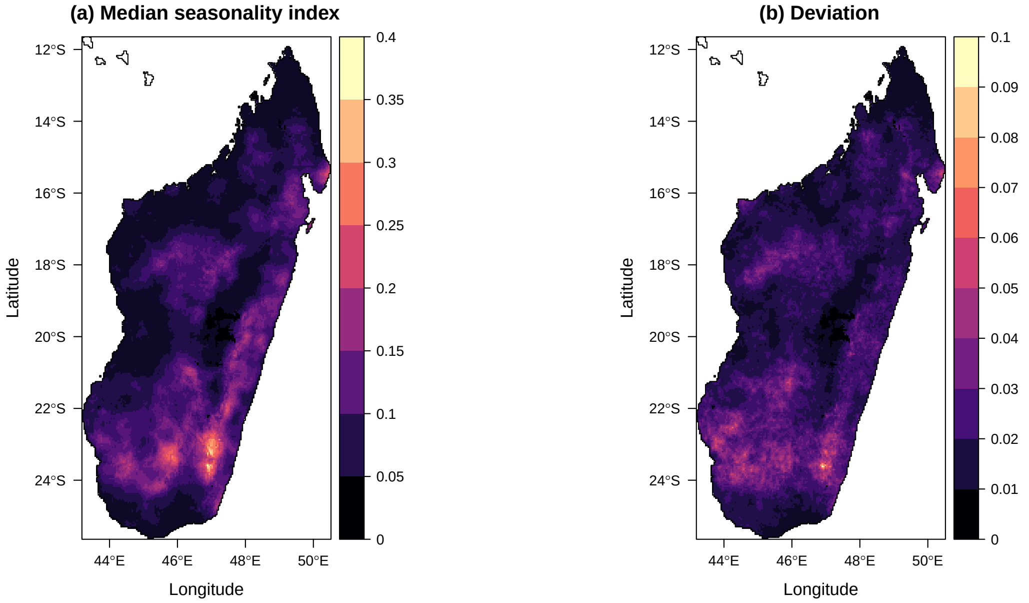

Figure S4: (a) Map of the median seasonality index in Madagascar and (b) the associated deviations.

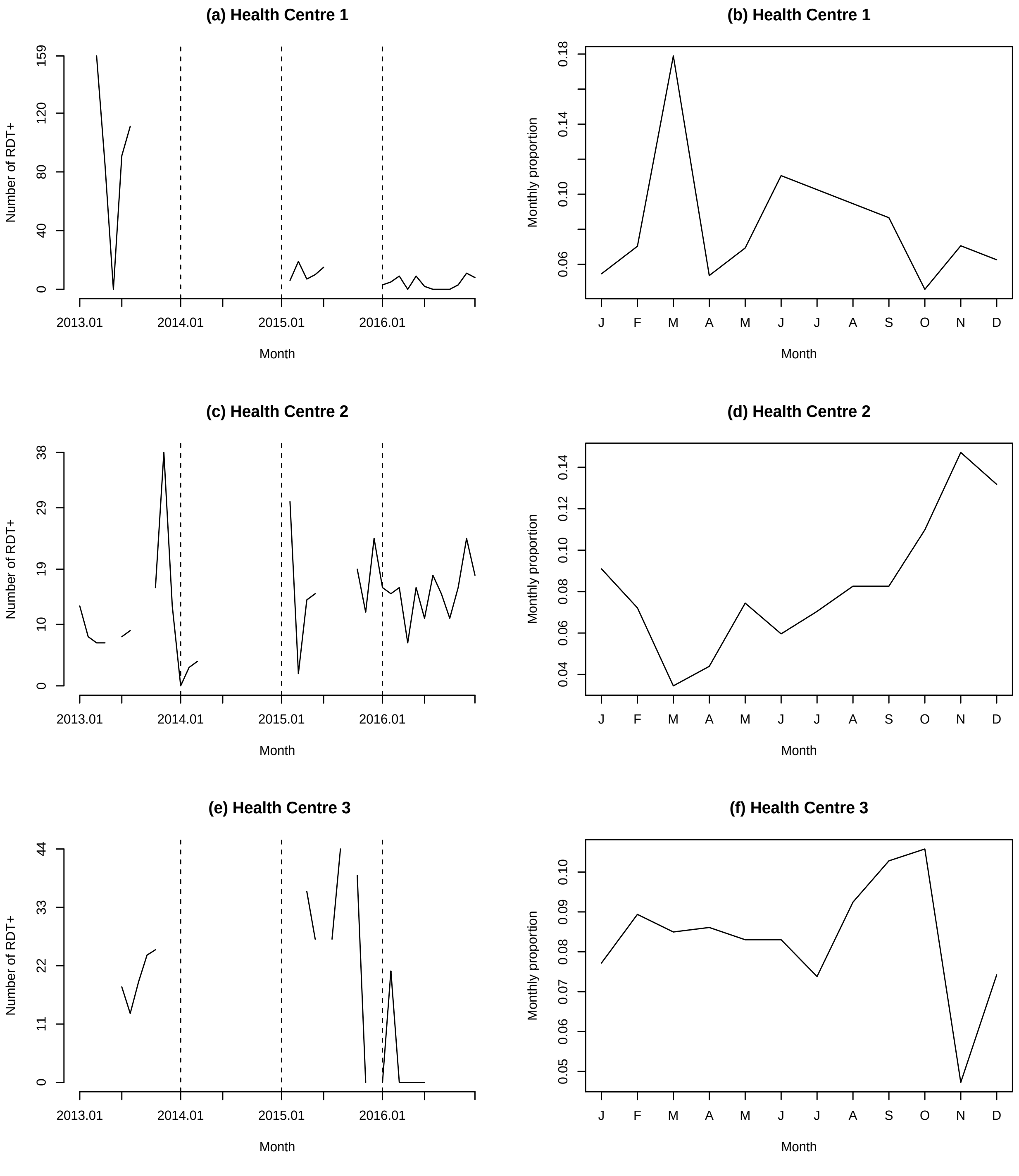

Figure S5: Number of positive rapid diagnostic tests (RDTs) recorded between 2013 and 2016 and the corresponding monthly proportions for three example health centres in Melaky.

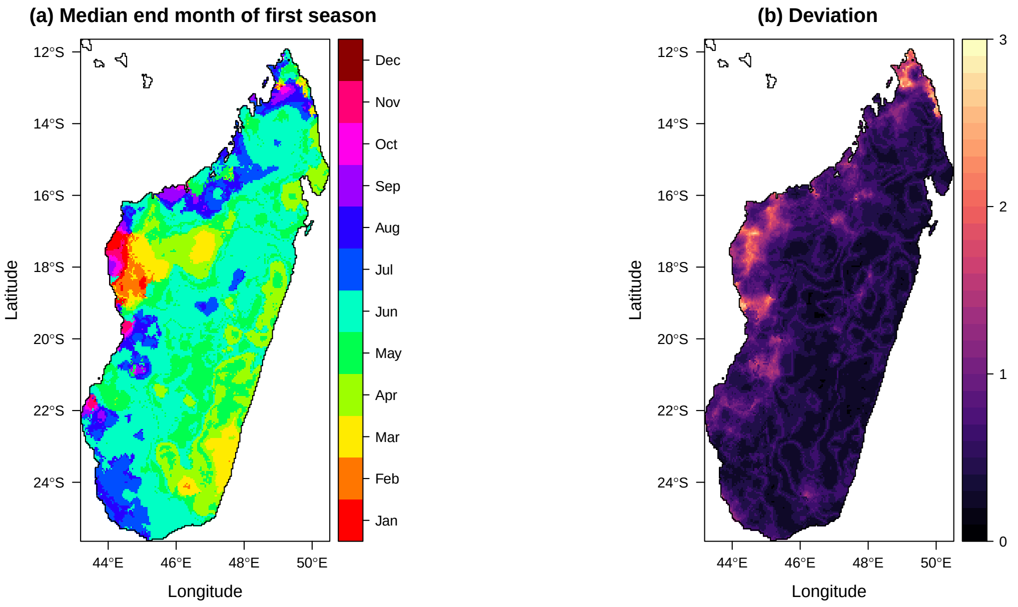

Figure S6: (a) Median end months of the first transmission season in Madagascar and (b) the associated deviations.

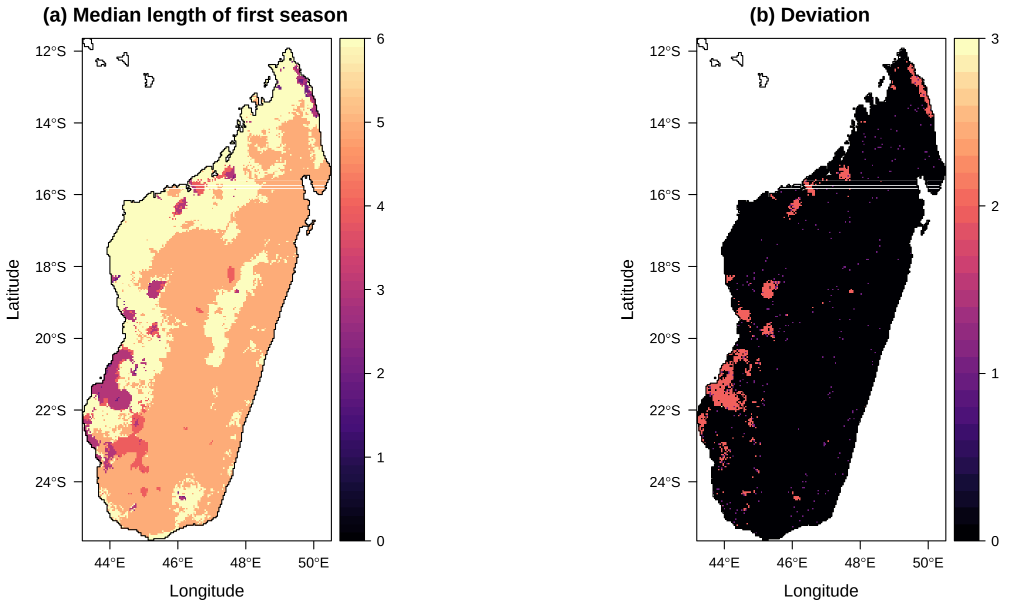

Figure S7: (a) Median length (in months) of the first transmission season in Madagascar and (b) the associated deviations.

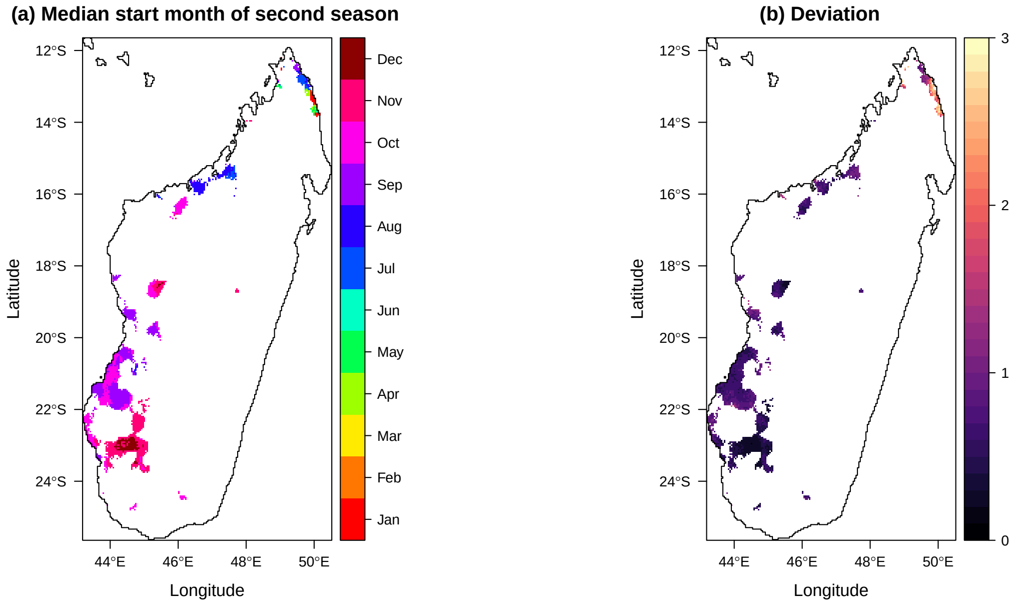

Figure S8: (a) Median start months of the second transmission season in Madagascar and (b) the associated deviations. Only the areas deemed as bimodal are coloured.

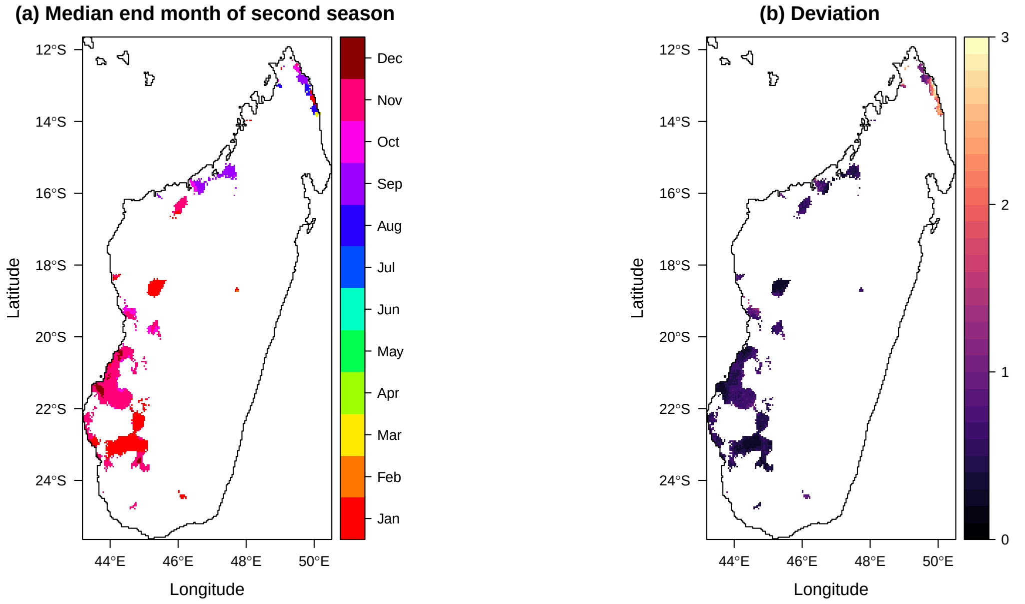

Figure S9: (a) Median end months of the second transmission season in Madagascar and (b) the associated deviations. Only the areas deemed as bimodal are coloured.

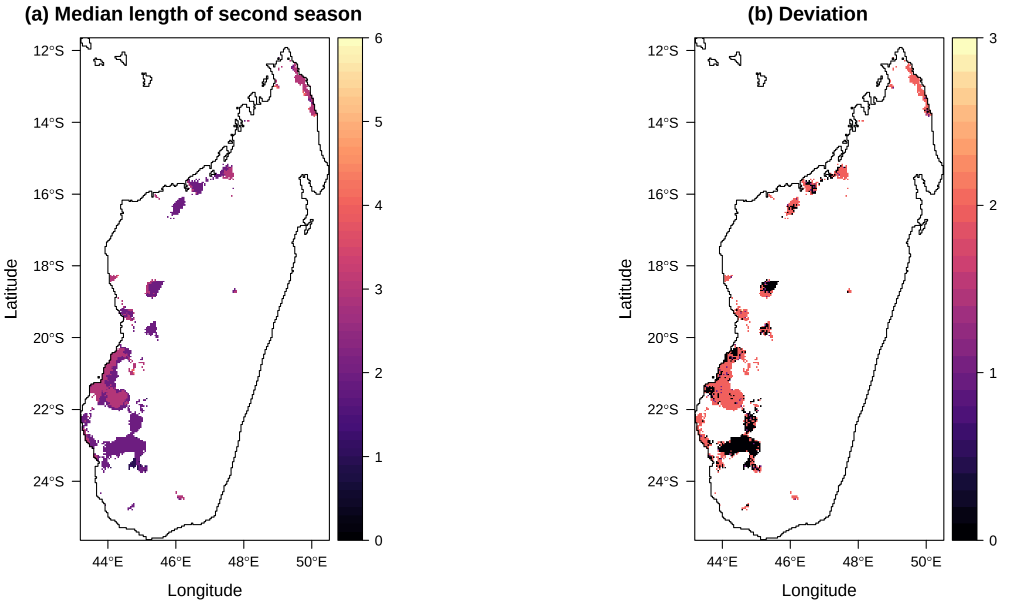

Figure S10: (a) Median length (in months) of the second transmission season in Madagascar and (b) the associated deviations. Only the areas deemed as bimodal are coloured.

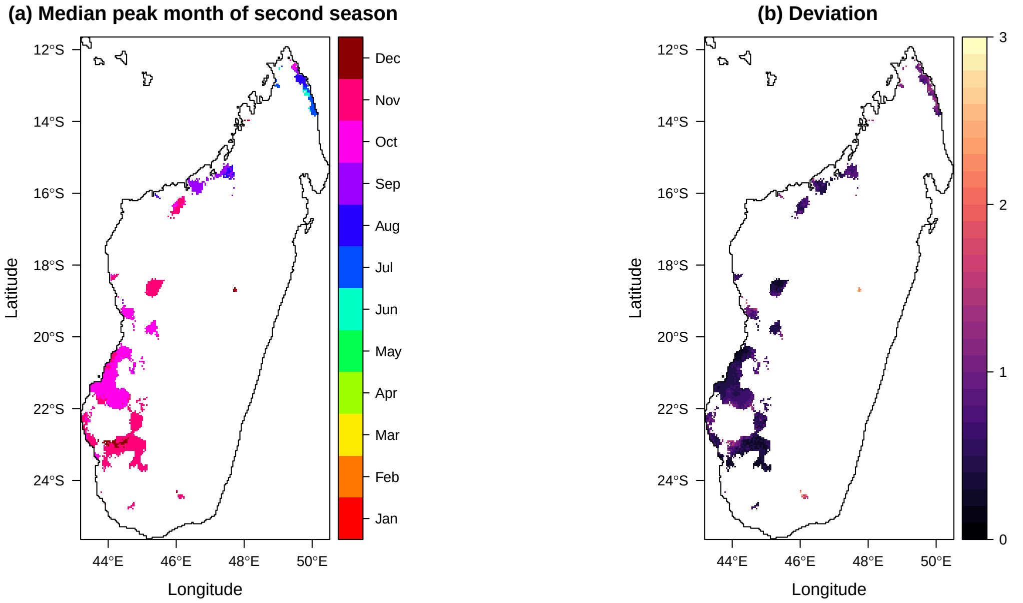

Figure S11: (a) Median peak months of the second transmission season in Madagascar and (b) the associated deviations. Only the areas deemed as bimodal are coloured.

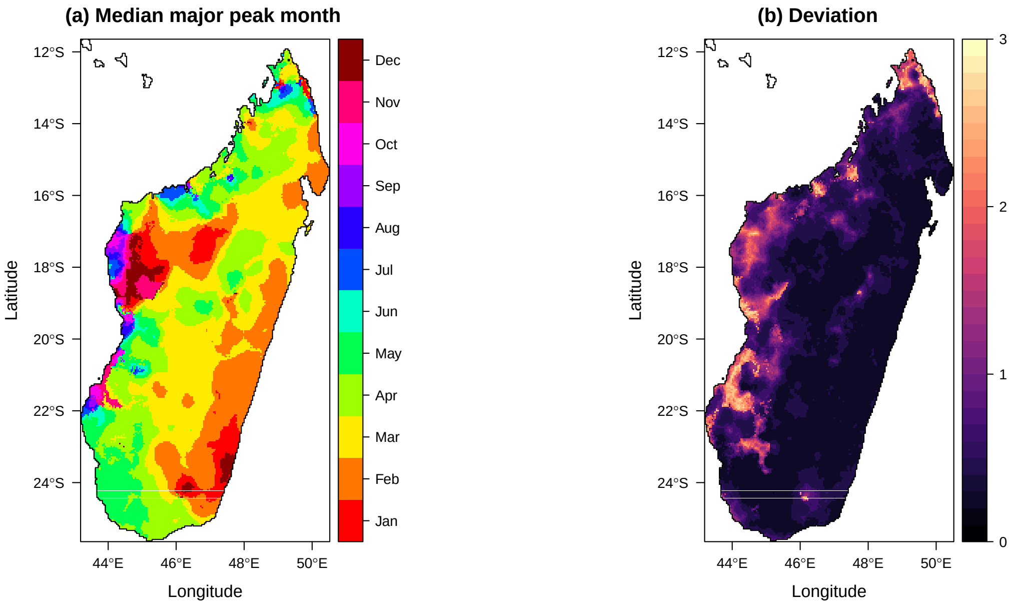

Figure S12: (a) Median major peak months in Madagascar and (b) the associated deviations. Note that this is the single peak for unimodal locations.

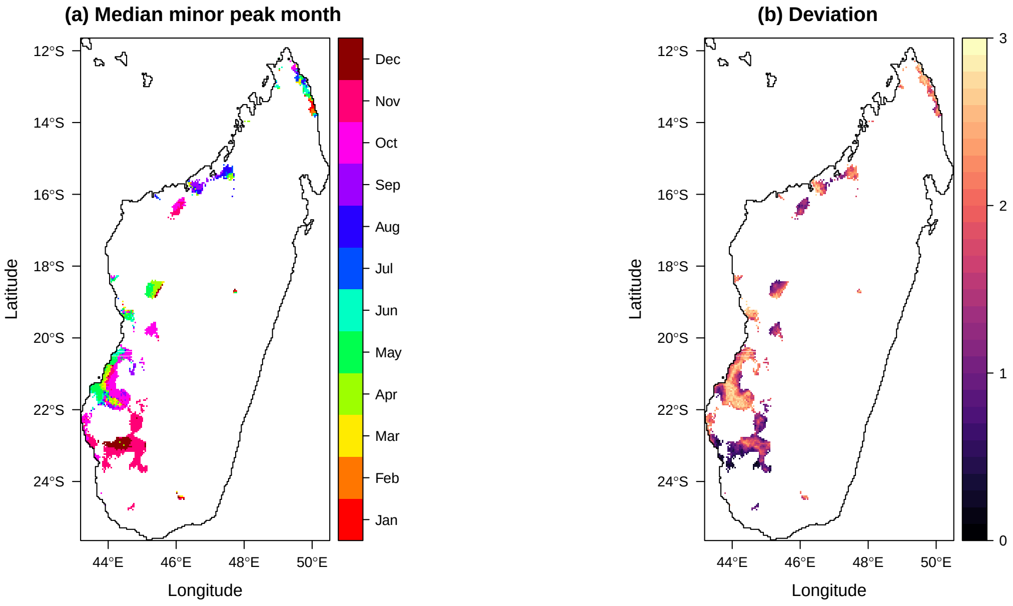

Figure S13: (a) Median minor peak months in Madagascar and (b) the associated deviations. This is only applicable for areas deemed as bimodal.

S2 Additional analysis

In this section, we investigate how the application of the methodology and the analysis results are affected by the quality and properties of the data including its spatial and temporal extents.

As shown in Fig. 4(a) and Fig. 5(a) in the main text as well as Fig. S6(a), spatial heterogeneity was observed for the estimated seasonal characteristics of Melaky. To understand this from the health facility data, we compute a measure of within-region synchrony by calculating the Pearson correlations between the empirical monthly proportions of the health facilities and that obtained by aggregating the cases in each region. Fig. S14 shows that Melaky has the lowest median correlation value and the largest range. This indicates that the observed seasonal patterns at the health facilities vary a lot within the region. As explained in the main text, this could be due to low and highly variable case numbers as well as reporting difficulties. In this way, the spatial heterogeneity in Melaky's empirical seasonal patterns led to the spatial heterogeneity in its estimated seasonality characteristics.

Since we compute monthly case proportions from the monthly case medians calculated across different years, the results of our analysis are highly dependent on the temporal extent of our data and how much seasonal information can be extracted. To illustrate this, we conduct two experiments: in the first, we remove data from 2013 and apply our methodology to the remaining data; in the second we remove data from 2013 and 2014. The model fitted to the full dataset from 2013-2016 achieved a high, positive Pearson correlation value of 0.767 between the estimated and empirical $\log(p_{i,j})$ values for all health facility locations. Compared to this baseline, the model chosen using data from 2014-2016 only gave a moderate but positive correlation of 0.385 while that chosen using data from 2015-2016 gave a correlation value of 0.311. The drop in performance of the chosen model can be related to how the temporal information is distributed across the years: 35.5% of the health facilities had more than 25% of their case data in 2013 while 32.7% of the health facilities had more than 25% of their data in 2014.

The proposed methodology is more robust towards changes in the spatial coverage of the Madagascar data than that of its temporal extent. When we reduce the number of health facilities from 2669 to 1401 and repeat our methodology, the Pearson correlation value between the estimated and empirical $\log(p_{i.j})$ values for all 2669 health facilities only reduces by 0.036 to 0.731. In addition, when we select our 30% test data so that they are clustered instead of randomly distributed in space, the correlation values remain relatively high at 0.729 when the test data are concentrated in the north-western part of the island and 0.730 when the test data are concentrated in the southern part of the island. Fig. S15 shows the locations of the test and training locations for these three scenarios. The ability to retain high correlation despite these changes in the spatial coverage of the data and the test set suggest that the dominant relationships between the seasonal patterns and the environmental covariates as well as the spatiotemporal correlation structures which were identified by the methodology are relatively strong and robust.

In the above analyses, we viewed the empirical monthly proportions obtained from the full 2013-2016 dataset as gold standard and computed Pearson correlation values with respect to them. This is because we were limited by the dataset which only contained 4 years of data. In general, the suitable length of the study period would depend on the study area. With climate change and ongoing malaria interventions, seasonal patterns could change over time. As such, one could use a moving window to analyse possible changes in seasonal patterns if longer study periods are available. Local knowledge and exploration of the data are also required to judge a suitable time-frame over which to characterise seasonal patterns.

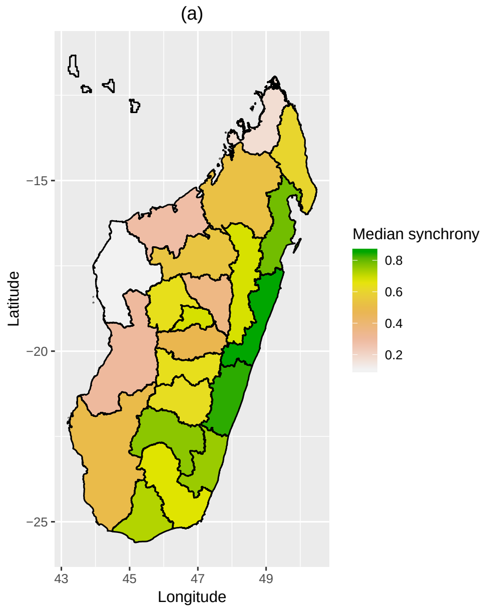

Figure S14: (a) Median synchrony measure and (b) the correponding variation or range for the regions in Madagascar. Synchrony is computed as the Pearson correlation between the empirical monthly proportions at a health facility and that obtained for the region after aggregating the positive cases from the available health facilities within the region.

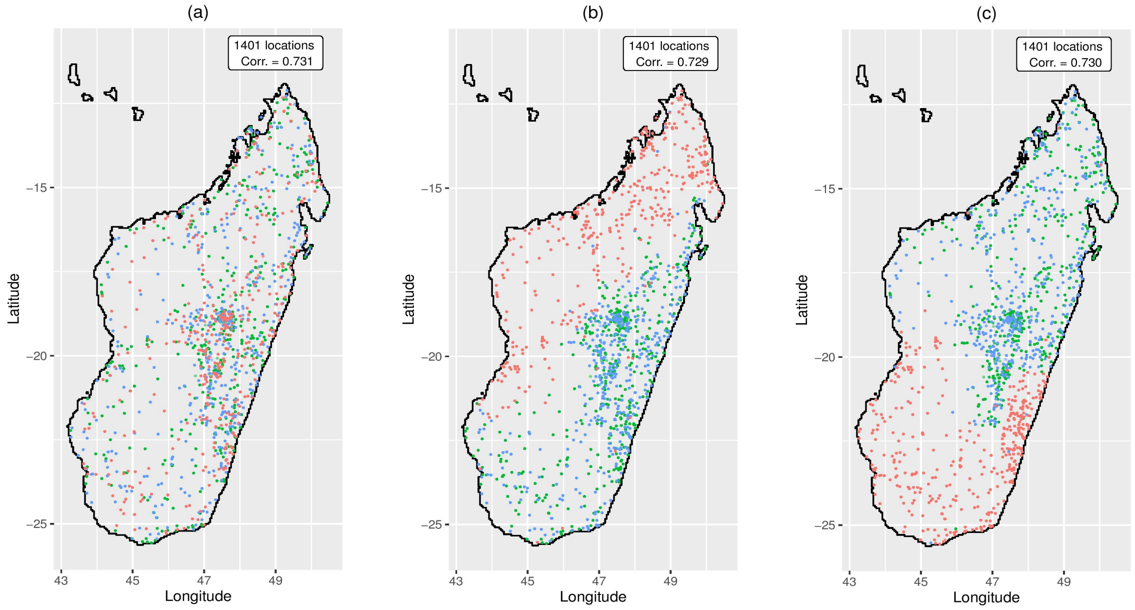

Figure S15: Locations of the test and training health facilities for Madagascar for the test scenarios with: (a) 1401 health facilities instead of 2669 health facilities; (b) the test set clustered in the north-western part of the island; and (c) the test set clustered in the southern part of the island. Here, the red, green and blue dots represent the test locations, training set 1 and training set 2 respectively.
